# Abstractness and social interaction through a new lens: the potentialities of hyperscanning in naturalistic settings

**DOI:** 10.3389/fnins.2025.1724845

**Published:** 2025-12-05

**Authors:** Ilenia Falcinelli, Chiara Fini, Anna M. Borghi

**Affiliations:** 1 Department of Dynamic and Clinical Psychology, and Health Studies, Sapienza University of Rome, Rome, Italy; 2 Institute of Cognitive Sciences and Technologies, Italian National Research Council, Rome, Italy

**Keywords:** abstractness, social interaction, hyperscanning, inter-brain synchronization, second-person neuroscience, learning, free conversations, naturalistic settings

## 1 The role of social interaction in acquiring and using abstract concepts

People constantly use concepts, allowing them to capture similar and different elements between objects and situations (Reilly et al., 2025), as mental shortcuts to organize information and navigate the inner and external world (Murphy, 2002; Villani et al., 2021).

Research has traditionally differentiated more concrete from more abstract concepts based on the reliance of the former on sensorimotor experiences (Paivio, 1990; Schwanenflugel et al., 1992). More abstract concepts typically lack a single physical referent and are more complex, heterogeneous, and variable in their meaning across people and contexts (Borghi and Mazzuca, 2023; Lewis et al., 2023). They are also usually acquired later than concrete ones, and this is mediated by language (e.g., Della Rosa et al., 2010; Wauters et al., 2003). Studies on language production (Brown, 1957, as reported in Schwanenflugel, 1991) and child-parent interaction (Bellagamba et al., 2022; Gillette et al., 1999) confirm that abstract concepts emerge later than concrete concepts; behavioral work also shows that emotional valence influences their acquisition (Lund et al., 2019; Ponari et al., 2018). In adults, most data show that abstract concepts are processed and recalled more poorly than concrete concepts (Concreteness effect, Paivio, 1990), likely due to their difficulty and variability. Research also testify that the distinction between abstract and concrete concepts may shift (Barsalou et al., 2018) in light of demographic characteristics, expertise, and context: for example, for older adults, technological concepts are more abstract than for younger adults (Falcinelli et al., 2024a), chemists consider H_2_O as less abstract than laypeople (Mazzuca et al., 2025a) and the concepts of gender is less abstractly characterized in a Dutch than an Italian sample (Mazzuca et al., 2024). Similarly, recent research highlights the multidimensional character of concepts: abstract concepts also evoke sensorimotor experiences, and each abstract concept kind, from emotions to numbers (Conca et al., 2021), is characterized by sensorimotor, linguistic, interoceptive, proprioceptive, and social experiences to varying degrees of significance (Borghi, 2022; Reilly et al., 2025).

In this opinion paper, we will focus on studies that highlight the role social interaction plays in abstract concepts' acquisition and use (Borghi et al., 2025).

The importance of social exchange for their acquisition is demonstrated across age groups: for instance, in children, extensive pacifier use, which might limit linguistic exchange, correlates with a less sharp abstract-concrete distinction (Barca et al., 2017), and adults report learning abstract concepts mainly through language (Falcinelli et al., 2024a,b; Villani et al., 2019).

The presence of others remains crucial for abstract concepts beyond first learning (Borghi et al., 2019; Dove et al., 2020; Fini and Borghi, 2019). Adults report less confidence in mastering abstract concepts and a higher need to rely on others to fully understand them (Falcinelli et al., 2024a,b; Fini et al., 2023; Mazzuca et al., 2022). In addition, in simulated conversations abstract topics lead to more expressions of uncertainty, questions, and need to continue the interaction (Villani et al., 2022), aspects which people also judge as more plausible for abstract concepts (Mazzuca et al., 2025b). Consistently, a preceding linguistic interaction on an abstract topic enhances to a greater extent participants' motor synchronization in a joint action task (Fini et al., 2021), and people are usually more open to negotiating abstract than concrete concepts' meaning (Falcinelli et al., 2024a; Fini et al., 2023).

Neuroscientific findings support these behavioral data, showing that, compared to more concrete ones, abstract concepts recruit to a greater extent brain networks including areas related to language and social cognition, such as the left inferior frontal gyrus (LIFG), the anterior temporal lobe (ATL), the left superior temporal sulcus/gyrus (LSTS/STG), and the left middle temporal gyrus (LMTG; for reviews, Borghi et al., 2019; Montefinese, 2019; for a meta-analysis, Wang et al., 2010).

The LIFG is typically associated with verbal working memory, semantic control, and executive regulation. Its higher activation for abstract concepts has been attributed to their greater variability and semantic complexity, which may require enhanced executive control, longer maintenance of abstract concepts in phonological short-term memory, and a more substantial involvement of inner speech (Binder et al., 2005; Della Rosa et al., 2018; Fernyhough and Borghi, 2023). Similarly, some evidence indicates that the LMTG, an area involved in semantic retrieval, is more strongly activated by abstract concepts, in line with their stronger association with linguistic rather than sensorimotor experience (Noppeney and Price, 2004). In addition, the ATL—commonly implicated in the semantic processing of auditory/verbal stimuli and according to some positions also in social cognition (Balgova et al., 2022; Visser and Lambon Ralph, 2011)—seems to show a stronger activation in its superior portion for abstract concepts, whereas in its ventromedial portion for concrete ones (Binney et al., 2016; Hoffman et al., 2015). Finally, some studies provide evidence that abstract concepts more greatly recruit the LSTS and STG, two regions involved in social cognition (Theory of Mind), along with auditory processing (Hoffman et al., 2015). According to some evidence, indeed, their activations and that of LIFG primarily depend on the (emotional-)social content of the provided stimuli (Mellem et al., 2016).

Despite the valuable insights coming from reviewed behavioral and neuroscientific studies, up to now evidence on the role of linguistic, social interactions for abstractness is mainly based on a “single-brain” approach (De Felice et al., 2023) where individuals are tested in isolation, for instance by asking them to evaluate concepts on semantic aspects (Falcinelli et al., 2024a,b; Fini et al., 2023; Mazzuca et al., 2022) or performing implicit tasks (e.g., semantic/lexical decision or judgment tasks) while recording cerebral activity (e.g., Della Rosa et al., 2018; Wang et al., 2018). Field research has recently moved toward more interactive—mainly conversational—paradigms, which allow researchers to explore communicative behaviors and indices of alignment between interlocutors (Fini et al., 2021; Mazzuca et al., 2025b; Villani et al., 2022). However, even these studies present some limits. First, experimental situations remain largely confined within the rigid setting of the laboratory. New positions are instead encouraging to “bring the laboratory to the real world,” to better account for the multiple and interrelated variables present in authentic situations (Vigliocco et al., 2024). Second, the reviewed interactive studies are mostly behavioral, neglecting other informative aspects like the online, synchronous neural processes that might occur between interagents. A complete understanding of the social mechanisms underlying abstract concepts' acquisition and use might therefore widely benefit from a more interactive neuroscientific perspective, inspired by the so-called “second-person neuroscience” (Konvalinka and Roepstorff, 2012), which allows for examining the neural coordination occurring during social interactions (Minagawa et al., 2018).

## 2 The promises of second person neuroscience and hyperscanning

“Second-person” neuroscience is a recent approach that goes beyond the traditional single-person perspective by studying brain activity during real-time social interactions (Redcay and Schilbach, 2019; Schilbach et al., 2013). Its key methodology is hyperscanning, consisting of the simultaneous recording of brain activity from two or more individuals (Montague et al., 2002). Hyperscanning can be implemented through different neuroimaging techniques, among which the most commonly used are EEG and fNIRS (Czeszumski et al., 2020). EEG enables to directly detect brain activity by recording neural oscillations through scalp electrodes, offering the advantage of capturing fast neural dynamics with a high temporal resolution (milliseconds; Czeszumski et al., 2020; Zamm et al., 2024); in contrast, fNIRS measures brain activity indirectly by assessing changes in blood oxygenation levels by detecting near-infrared light absorption. Although fNIRS provides slower (hemodynamic) data, it is more resistant to motion artifacts and allows for a better spatial localization (Cui et al., 2012; Czeszumski et al., 2020). Through these modalities, hyperscanning enables to capture the Inter-Brain Synchronization (IBS)—i.e., the temporal alignment or correlation of neural activity patterns between people (Montague et al., 2002)—, which represents an index of the harmonization in underlying neural processes (Kelsen et al., 2022). Mechanistically, IBS arises when participants' neural oscillations or hemodynamic responses become temporally entrained during social exchanges, potentially driven by reciprocal sensorimotor and cognitive feedback. The quantification of IBS can be extracted from several indices: EEG-based measures include *inter-brain phase coherence, phase-locking value*, and *phase locking index*, capturing consistent phase relationships in neural oscillations across brains (Gugnowska et al., 2022; Müller and Lindenberger, 2023); fNIRS analyses instead typically use coherence or correlation of oxygenated and deoxygenated hemoglobin signals between homologous regions of interacting participants, allowing the detection of harmonic hemodynamic patterns related to social behaviors (Cui et al., 2012; Fishburn et al., 2018).

The advent of the portable version of EEG and fNIRS devices, which reduce physical constraints, has further extended hyperscaning possibilities, allowing researchers to perform more realistic or outdoor studies with ease (Astolfi et al., 2011).

Also due to this, hyperscanning research has increasingly expanded around the globe (Grasso-Cladera et al., 2024), to investigate the “social brain” during various “life-skills” like cooperation, competition, and communication (e.g., Astolfi et al., 2020; Balconi and Vanutelli, 2017, 2018; Ahn et al., 2018), embedded in several situations such as knowledge sharing (e.g., Bevilacqua et al., 2019; Dikker et al., 2017; Pan et al., 2020; Zhu et al., 2022), naturalistic conversations (e.g., Ahn et al., 2018; Kinreich et al., 2017; Kustova et al., 2023; Pérez et al., 2019), work reviews (e.g., Balconi et al., 2020), clinical interventions (e.g., Pan and Cheng, 2020; Zhang et al., 2018), musical synchronization (e.g., Gugnowska et al., 2022; Müller and Lindenberger, 2023), playing games (e.g., Astolfi et al., 2009; Jahng et al., 2017), or simply resting state situations (e.g., Kustova et al., 2023; Zheng et al., 2020).

In our view, hyperscanning research focusing on spoken language—specifically, on knowledge sharing and naturalistic discussions—may be particularly beneficial for investigating the role of social exchanges for abstractness, as we discuss below.

## 3 Hyperscanning studies on knowledge sharing and their potentialities for investigating abstract concepts' acquisition

Hyperscanning research has extensively investigated knowledge sharing in real classroom contexts, focusing on synchronous brain dynamics occurring between teacher and students (De Felice et al., 2023; Kelsen et al., 2022; Tan et al., 2023, for reviews).

A central finding is that interactive learning—such as active discussions, scaffolding questions, video usage, or personalized feedback—fosters greater teacher-student and student-student IBS than traditional lecture-based methods (Dikker et al., 2017; Pan et al., 2020).

In this context, IBS has been mainly found in prefrontal and temporo-parietal regions (Bevilacqua et al., 2019; Dikker et al., 2017; Pan et al., 2020)—areas usually associated with social cognition, mentalizing system, and linguistic abilities (Kelsen et al., 2022)—with greater IBS in the left dorsolateral prefrontal cortex (dlPFC) at increased teachers' expertise (Sun et al., 2020), and higher IBS in the right temporo-parietal junction (Zheng et al., 2018) and in the sensorimotor and left parietal cortices (Zheng et al., 2020; Zhu et al., 2022) at increased interactivity of pedagogical techniques.

Crucially, enhanced IBS correlates with improved learning outcomes (Pan et al., 2020; Zheng et al., 2018). For example, greater expert-student IBS in the dlPFC during cooperative learning aligns with performance gains (Sun et al., 2020). Similarly, segmented, interactive musical teaching increases IBS in the bilateral inferior frontal cortex and correlates with better skill acquisition (Pan et al., 2018).

Taken together, these findings suggest that social, interactive learning induces stronger teacher-student neural coupling and better learning outcomes. Given that abstract concepts greatly rely on linguistic and social input for their acquisition, we contend that translating this approach into categorization research—for instance, by investigating parent-infant interaction for basic concepts or expert-layperson exchanges for scientific concepts, both face-to-face and technologically-mediated/supported—could significantly deepen the understanding of the social underpinnings of abstract concepts' acquisition.

## 4 Hyperscanning studies on natural discussions and their potentialities for investigating abstract concepts' use

Hyperscanning research has expanded beyond structured learning to explore brain-to-brain coupling during authentic verbal interactions, often using portable fNIRS or EEG for more ecological validity (Kelsen et al., 2022, for a review). Consistent with prior findings (Section 3), higher IBS emerges mainly in frontal and temporal-parietal regions. For example, Jiang et al. (2012) showed increased IBS in the left inferior frontal cortex during face-to-face dialogues compared to monologs or back-to-back talks.

Integrating these findings, some studies suggest that several factors modulate IBS during free interactions: among them, social bounds—romantic couples exhibit stronger temporal-parietal synchrony than strangers (Kinreich et al., 2017)—, employed language—different IBS patterns (i.e., more concentrated vs. more distributed) emerge in fronto-central areas during conversations in native versus second languages (Pérez et al., 2019)—and type of interaction setting—for instance, in simulated counseling sessions, greater counselor-client IBS in the right temporal-parietal junction was associated with a stronger therapeutic alliance (Zhang et al., 2018); similarly, increased IBS in frontal regions was found between a manager and an employee during a non-rating than rating simulated performance review (Balconi et al., 2020).

Furthermore, some EEG and MEG studies focusing on turn-taking—a pillar of conversation—reveal significant speaker-listener phase synchronization (in alpha, gamma, and theta bands) within frontal and temporal-parietal areas, suggesting the presence of neural mechanisms supporting speech coordination (Ahn et al., 2018; Kawasaki et al., 2013; Pérez et al., 2017). Crucially, preliminary data show that informal free dialogue between mentor and mentee fosters higher IBS (in theta, alpha, and beta bands) than structured work discussions, indicating that more reciprocal and flexible interactions promote greater neural synchrony (Kustova et al., 2023).

Collectively, these findings suggest that neural coupling mechanisms support coordination and mutual understanding in free communication, with higher IBS observed in less structured conversation formats. This body of research provides a promising avenue for investigating the significance of social interaction when using abstract concepts. Given that abstract concepts are more variable (Section 1), the neural coupling captured via hyperscanning in naturalistic conversations can illuminate how interlocutors refine, negotiate, and co-construct their meaning in real-time, also considering some impacting aspects like the kind of bond, the interactive situation (e.g., symmetric vs. asymmetric) and the main interaction style (imitating vs. complementary) of interlocutors.

## 5 Conclusions

Since abstract concepts are more complex and variable than concrete ones, the presence of others is fundamental for their acquisition and use. Current field studies, however, typically employ a “single-brain” approach or, when more interactive, they rely only on behavioral data collected in laboratory settings.

The emerging field of second-person neuroscience, via hyperscanning, offers a promising approach to addressing these limitations. Hyperscanning simultaneously records the brain activity of multiple interacting individuals, capturing the online IBS occurring among them. IBS has been consistently found in frontal and temporal-parietal regions, which are linked to social cognition, mentalizing, and language, across several real-time situations like knowledge sharing and naturalistic conversations. In our view, the integration between these two fields may enhance categorization research: knowledge-sharing research can illuminate the neural mechanisms underlying abstract concept acquisition; naturalistic conversation research can deepen our understanding of how interlocutors' brains align when they co-construct and negotiate abstract concepts' meanings. This integration might also represent a unique opportunity to extend the existing single-brain neuroscientific findings by investigating whether linguistic and social critical regions (i.e., LIFG, ATL, LSTS/STG, and LMTG) synchronize more across brains during social exchanges on abstract than concrete concepts. Portable hyperscanning technologies can assist these explorations, by facilitating studies in more ecological settings.

Despite these advantages, hyperscanning research is still facing significant technical and theoretical challenges (Zamm et al., 2024). These include its inherently correlational nature that limits causal inference (Novembre and Iannetti, 2021), the risk that observed IBS may be a simple reflection of common sensorimotor inputs rather than the expression of high-level alignment processes (Hamilton, 2021; Holroyd, 2022), the current absence of standardized theoretical frameworks and measurement methods (Holroyd, 2022; see also Section 2) and also practical set-up issues, such as ensuring a precise synchronization between brain recordings and stimuli presentation across interacting individuals (Barraza et al., 2019; for other technical issues, see Zamm et al., 2024). Addressing these aspects requires rigorous and standardized operational definitions, experimental set-ups, methodologies, and data pipelines (for examples see Chidichimo et al., 2025; Kayhan et al., 2022; Zimmermann et al., 2024).

In addition, like single-brain studies, hyperscanning research can suffer from the intrinsic constraints of the employed methodologies. For instance, although fNIRS provides better spatial resolution than EEG, it is restricted to cortical surface activity due to the shallow penetration depth of near-infrared light (Czeszumski et al., 2020; Zamm et al., 2024). This aspect might limit the investigation of the role of critical but deeper brain structures—such as the ATL—in shared dynamics occurring during abstract concepts' acquisition and use. Fortunately, hyperscanning research could draw upon solutions found for similar challenges in other fields (e.g., development of optimized acquisition sequences to reduce signal dropout in fMRI studies—Embleton et al., 2010; Halai et al., 2014, 2024), or, at least, benefit from the integration of multiple imaging and bodily measures within the same study (Grasso-Cladera et al., 2024).

In conclusion, by taking proper precautions, translating current hyperscanning research on spoken language into the field of categorization could yield interesting insights into how abstract concepts are acquired and used in the presence of others, thereby enriching categorization research.
